# Supplementary material for: Elevated Expression of miR-200c/141 in MDA-MB-231 Cells Suppresses MXRA8 Levels and Impairs Breast Cancer Growth and Metastasis In Vivo
Source: Genes (Basel). 2022 Apr 14;13(4):691. doi: 10.3390/genes13040691 (PMC9032019; doi:10.3390/genes13040691)
Supplement: Supplementary file 1 [file genes-13-00691-s001.zip › genes-1652332-supplementary.pdf]

Supplemental File S1

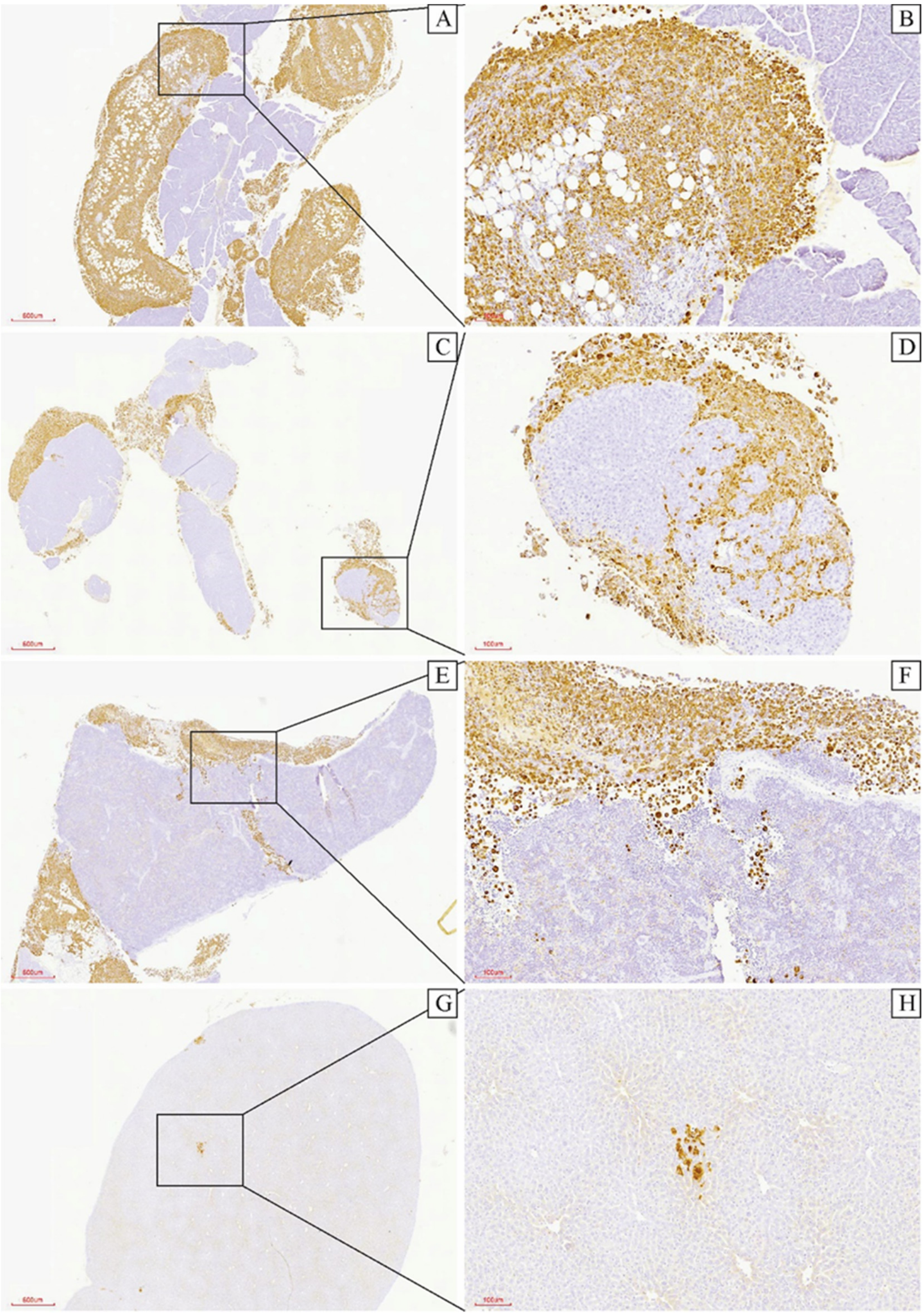

Figure S1. Vimentin stained metastatic MDA-231EV cells in (**A–D**) pancreas, (**E**,**F**) spleen, and (**G**,**H**) liver. Metastatic tumor cells were frequently found near the (**A**,**B**) pancreas and (**E**,**F**) spleen and (**C**,**D**) only rarely invaded the pancreas. Scale bars for A,C,E,G are 600 _m and for B,D,F,H are 100 μm.

Supplemental File S2

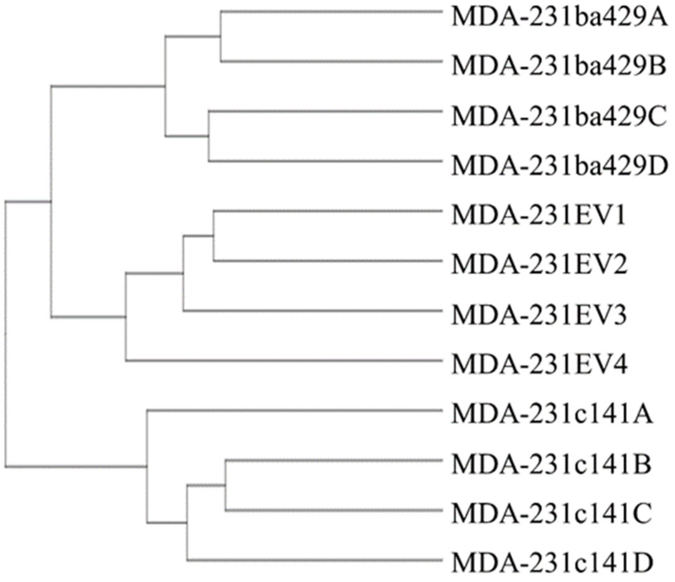

Figure S2. Hierarchical clustering of MDA-231EV, MDA-231c141 and MDA-231ba429 tumors.

Supplemental File Table S1

Table S1. Transcripts with miR-200c or miR-141 binding sites.

**Transcript**			
	**miRDB**	**MicroT-CDS**	**miRwalk**
CPE	-	-	miR-200c-5p
GNG2	-	-	miR-141-3p, miR-141-5p, miR-200c-5p
MXRA8	-	-	miR-141-5p, miR-200c-3p
SELENBP1	-	-	miR-141-5p, miR-200c-3p, miR-200c-5p
AGR2	-	-	miR-141-5p
JAG1	miR-141-3p	miR-141-3p	miR-141-3p, miR-141-5p, miR-200c-3p, miR-200c-5p
LCP1	-	-	miR-141-3p, miR-141-5p, miR-200c-3p
DYSF	-	-	miR-141-3p, miR-141-5p, miR-200c-3p
NKD1	miR-141-3p	-	miR-141-5p, miR-200c-5p
CALHM2	-	-	miR-200c-5p
AFAP1-AS1	Not found	Not found	Not found
RAB9A	-	miR-141-5p	miR-141-5p, miR-200c-3p
EDNRA	-	miR-200c-3p	miR-141-3p, miR-141-5p, miR-200c-3p, miR-200c-5p
FSIP2	Not found	-	miR-141-5p, miR-200c-5p
SERPINF1	-	-	miR-200c-3p
